# Prevalence of inflammatory bowel disease in two districts of Sri Lanka: a hospital based survey

**DOI:** 10.1186/1471-230X-10-32

**Published:** 2010-03-19

**Authors:** Madunil A Niriella, Arjuna P De Silva, Asangi HGK Dayaratne, Madurangi HADP Ariyasinghe, Metthanandha MN Navarathne, Ranjith SK Peiris, D Nandadeva Samarasekara, Raveendra L Satharasinghe, Sharman Rajindrajith, Anuradha S Dassanayake, A Rajitha Wickramasinghe, H Janaka de Silva

**Affiliations:** 1 University Medical Unit, Colombo North Teaching Hospital, Ragama, Sri Lanka; 2 Department of Medicine, Faculty of Medicine, University of Kelaniya, Ragama, SriLanka; 3 Gastroenterology Unit, National Hospital of Sri Lanka, Colombo, Sri Lanka; 4 Gastroenterology Unit, Colombo South Teaching Hospital, Kalubowila Sri Lanka; 5 Department of Surgery, Faculty of Medicine, University of Colombo Sri Lanka; 6 Medical Unit, Sri Jayawardenapura General Hospital, Kotte, Sri Lanka

## Abstract

**Background:**

Inflammatory bowel disease (IBD) is being increasingly diagnosed in Asia. However there are few epidemiological data from the region.

**Methods:**

To determine prevalence and clinical characteristics of IBD, a hospital-based survey was performed in the Colombo and Gampaha districts (combined population 4.5 million) in Sri Lanka. Patients with established ulcerative colitis (UC) and Crohn's disease (CD), who were permanent residents of these adjoining districts, were recruited from hospital registries and out-patient clinics. Clinical information was obtained from medical records and patient interviews.

**Results:**

There were 295 cases of IBD (UC = 240, CD = 55), of which 34 (UC = 30, CD = 4) were newly diagnosed during the study year. The prevalence rate for UC was 5.3/100,000 (95% CI 5.0-5.6/100,000), and CD was 1.2/100,000 (95% CI 1.0-1.4/100,000). The incidence rates were 0.69/100,000 (95% CI 0.44-0.94/100,000) for UC and 0.09/100,000 (95% CI 0.002-0.18/100,000) for CD. Female:male ratios were 1.5 for UC and 1.0 for CD. Mean age at diagnosis was (males and females) 36.6 and 38.1y for UC and 33.4 and 36.2y for CD. Among UC patients, 51.1% had proctitis and at presentation 58.4% had mild disease. 80% of CD patients had only large bowel involvement. Few patients had undergone surgery.

**Conclusions:**

The prevalence of IBD in this population was low compared to Western populations, but similar to some in Asia. There was a female preponderance for UC. UC was mainly mild, distal or left-sided, while CD mainly involved the large bowel.

## Background

Inflammatory bowel disease (IBD) includes ulcerative colitis (UC) and Crohn's disease (CD), which are chronic inflammatory diseases of the gastrointestinal tract. The underlying aetiology and pathogenesis of IBD remains largely unknown, but are thought to result from an interaction between genetic susceptibility, environmental factors and the host immune response. IBD has been regarded as a disease primarily occurring in Western populations. High prevalence rates have been reported from North America, United Kingdom, and Northern Europe [1-3]. Although it was earlier considered rare in Asia [4-6], a number of recent reports suggest an increase in the incidence and the prevalence of IBD in the region [7-13]. Although better diagnostic awareness and increasing access to modern endoscopic technology may play a role, these factors alone are unlikely to explain this increase. Reasons for the increasing incidence of IBD in Asia are probably multi-factorial, and may at least partly be related to development and prosperity, resulting in improved hygiene and changing dietary habits [14]. Investigating the early stages of IBD as it emerges in new populations may provide a new opportunity to study its pathophysiology.

Unfortunately, data on the epidemiology and clinical characteristics of IBD in many Asian countries, and for that matter in most developing countries, are scarce [13]. Epidemiological and clinical information on IBD in many of these countries suffer from poor reporting networks, limited population based data, hospital data from undefined catchment areas, and non-uniform diagnostic criteria. There are also problems in diagnosis due to lack of awareness, poor resources, and problems of differentiating IBD from intestinal infections; circumstances where accurate incidence and prevalence figures are difficult to derive [14].

Sri Lanka is a country in economic transition, and disease patterns reflect this; there has been a gradual decline in diseases associated with poverty and an increase in non-communicable diseases. The epidemiology of IBD in Sri Lanka has not been investigated. We conducted a study to determine the prevalence and clinical characteristics of IBD in the Colombo and Gampaha districts of Sri Lanka. These two adjoining districts were selected as they appeared to be best suited for a hospital based survey: the two districts have a well defined catchment area for the regional hospitals; they are the most developed and populous in Sri Lanka; they have a reasonable ethnic and urban-rural mix, and high literacy rates (>95%) which would positively impact on health seeking behaviour [15].

## Methods

### Study population

**Figure 1:**
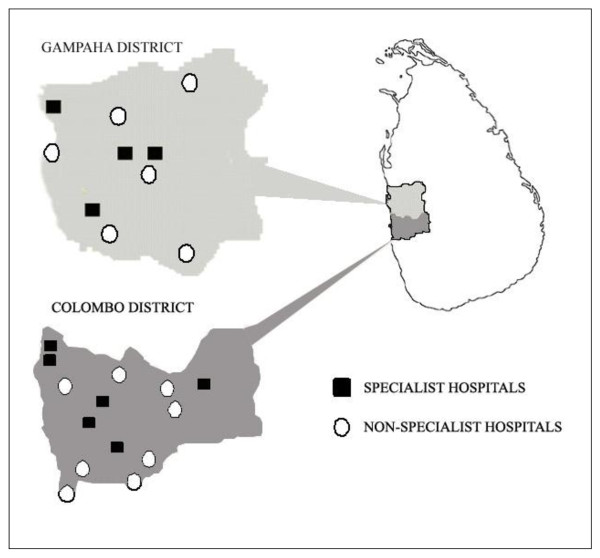
**Map of Sri Lanka showing the locations of specialist and non-specialist hospitals in the Colombo and Gampaha districts**.

A hospital-based survey was performed over a period of one year from December 2007 to November 2008 in the Colombo and Gampaha districts of Sri Lanka. The combined population of the two districts is approximately 4.5 million [14], and is served by 24 state sector hospitals (that offer free health care); 10 offering specialist services and 14 that do not (Figure 1). As the most urbanised and developed districts with the highest level of health care in the country it can be assumed that the population of these adjoining districts is stable.

### Sampling and subject recruitment

A preliminary survey, by telephone interview, was conducted among Medical Officers-in-Charge of the 14 hospitals in the two districts which do not offer specialist services. Patients suspected to have IBD had been encountered in 5 of these 14 hospitals. However, none of the non-specialist hospitals treated or followed-up IBD patients, and always referred suspected cases to the 10 specialist hospitals. Therefore, only the 10 specialist hospitals in the two districts were included in the hospital survey.

Patient registries in the selected hospitals were examined, and patients with established IBD who were permanent residents in the two districts were recruited. The diagnosis of IBD had to satisfy accepted clinical, radiological and endoscopic criteria with histological confirmation [16]. Patient identities were cross-checked to avoid replication. Clinical information was obtained by both review of case records and patient interviews after written informed consent. The following information was obtained: demographic characteristics, age at diagnosis, clinical presentation, duration of disease, family history of IBD, extent (assessed by colonoscopy, small bowel studies, and histology) and severity (assessed by endoscopy and histology according to the opinion of the investigators) of the disease at the time of diagnosis, presence of extra-intestinal manifestations, treatment modalities and the requirement for surgical intervention. The anatomical extent of UC as determined by the first-time colonoscopy was classified as distal colitis (limited to the rectum and sigmoid colon), left-sided colitis (disease distal to the splenic flexure) or "extensive" colitis (extending proximal to the splenic flexure) [17]. The extent of CD, was determined by endoscopic and radiological methods, and was classified as upper gastrointestinal, small bowel, large bowel and ano-rectal disease.

The population figures and other demographic data for the two districts were obtained from the Department of Census and Statistics of Sri Lanka [15]. The prevalence rates of UC and CD were calculated using the total number of residents in the two districts (catchment area) as the denominator and the total number of patients with UC and CD respectively as the numerators. The incidence rates were calculated using the number of new cases diagnosed during the year of the study as the numerators.

### Statistical analysis

The crude prevalence and incidence rates for UC and CD were calculated. The rates were expressed as number of patients per 100,000 population. Ninety five percent confidence intervals (95% CI) for prevalence and incidence rates were estimated.

### Ethics

Ethical approval for the study was obtained from the Ethical Review Committee of the Faculty of Medicine, University of Kelaniya.

## Results

**Figure 2:**
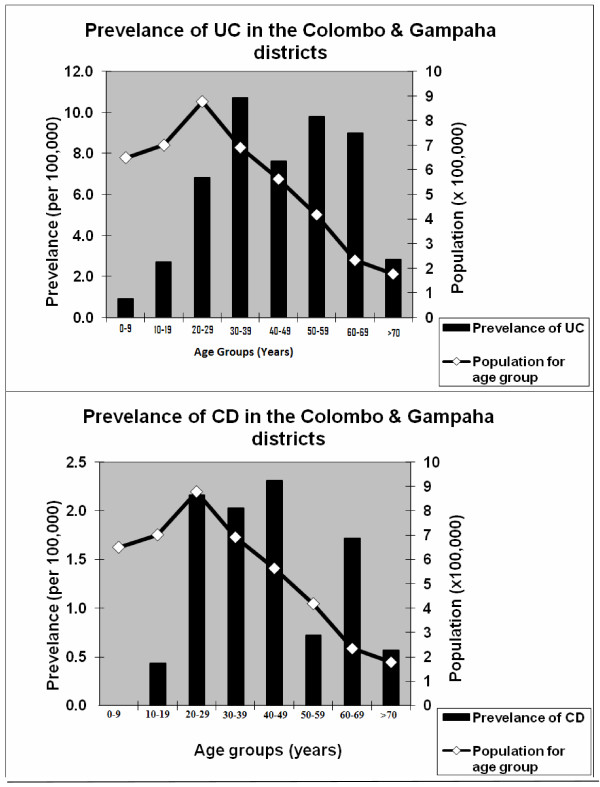
**Age-specific prevalence of IBD in the Colombo and Gampaha districts**.

There were 295 confirmed cases of IBD (UC = 240, CD = 55). Of these, 34 cases (UC = 30, CD = 4) were newly diagnosed during the study year. The prevalence of UC was 5.3/100,000 (95% CI 5.0-5.6/100,000) and CD was 1.2/100,000 (95% CI 1.0-1.4/100,000). The incidence of UC was 0.69/100,000 (95% CI 0.44 - 0.94/100,000) and CD was 0.09/100,000 (95% CI 0.002-0.18/100,000). Age related prevalence for UC and CD are given in Figure 2. Among 295 confirmed cases of IBD, the female:male ratios for UC and CD were 1.5 and 1.0 (Table 1). This trend for female preponderance in UC was observed in different age groups except in the 15 to 19 year group (Table 2). Of patients with UC, 89.7% were Sinhalese, 7.3% Tamil and 3% Muslim. 90.9% with CD were Sinhalese, 7.3% Tamil and 1.8% Muslim. The observed differences in type, extent and severity of disease generally reflected the districts' ethnic demography (Table 3). Mean age at diagnosis (males and females) for UC was 36.6 and 38.1 years, and for CD 33.4 and 36.2 years.

**Table 1:** Clinical characteristics of IBD patients

	Ulcerative colitis (UC) (N = 240)	Crohn's disease (CD) (N = 55)
**Sex**		
**Females**	144	28
**Males**	96	27

**Mean age at diagnosis**		
**Females**	38.1	33.4
**Males**	36.6	36.2

**Ethnicity**		
**Sinhalese**	209 (89.7%)	50 (90.9%)
**Tamil**	17 (7.3%)	4 (7.3%)
**Muslim**	7 (3%)	1 (1.8%)
	-	-

**Extent of disease (UC-221)**		
**Distal**	113 (51.1%)	-
**Left-sided**	50 (22.6%)	-
**Extensive**	58 (26.3)	-
		
**Extent of disease (CD-55)**		
**Only small intestinal involvement**	-	7 (12.7%)
**Only large intestinal involvement**	-	44 (80%)
**Large & small intestinal involvement**	-	4 (7.3%)
**Perianal disease**	-	4 (7.3%)
**Upper gastrointestinal involvement**	-	3 (5.5%)

**Severity of disease**	**(UC-166)**	**(CD-45)**
**Mild**	97 (58.4%)	26 (57.7%)
**Moderate**	49 (29.5%)	10 (22.2%)
**Severe**	20 (12.2%)	9 (20%)

**Extra-intestinal manifestations**		
**Arthralgia and arthritis**	68 (28.3%)	13 (23.6%)
**Ocular disease**	22 (9.2%)	9 (16.4%)
**Skin rashes**	18 (7.5%)	3 (5.5%)
**Backache**	17 (7.1%)	3 (5.5%)

**Positive family history**	5 (2.1%)	3 (5.5%)

**Surgical intervention**	11 (4.6%)	10 (18.2%)

**Table 2:** Gender differences (expressed as Female:Male ratios) among IBD patients in different five-year age groups

Age group (years)	UC (F:M ratio/100,000)	CD (F:M ratio/100,000)
00-04	-	-
05-09	1.50	-
10-14	2.00	-
15-19	0.71	0.33
20-24	1.44	0.83
25-29	1.36	1.66
30-34	1.76	1.50
35-39	1.73	0.75
40-44	1.38	1.33
45-49	1.50	1.33
50-54	1.44	0.00
55-59	1.66	1.00
60-64	1.20	2.00
65-69	1.50	0.00
70-74	1.00	0.50
> 75	-	-

**Table 3:** The extent and severity of IBD among different ethnic groups

	Sinhala	Tamil	Muslims
General population of the two districts*	83.5%	8%	6.5%

**UC**	209 (89.7%)	17 (7.3%)	7 (3%)

**Extent of UC**			

Distal	103 (91.2%)	7 (6.2%)	3 (2.6%)

Left sided	43 (86%)	4 (8%)	3 (6%)

Extensive	51 (88%)	6 (10%)	1 (2%)

**Severity of UC**			

Mild	90 (92.8%)	4 (4.1%)	3 (3.1%)

Moderate	48 (98%)	-	1 (2%)

Severe	18 (90%)	2 (10%)	-

**CD**	50 (90.9%)	4 (7.3%)	1 (1.8%)

**Extent of CD**			

Only small intestinal involvement	6 (85.7%)	1 (14.3%)	-

Only large intestinal involvement	41 (93.2%)	2 (4.5%)	1 (2.3%)

Large and small intestinal involvement	3 (75%)	1 (25%)	-

Perianal disease	3 (75%)	-	1 (25%)

Upper gastrointestinal involvement	3 (100%)	-	-

**Severity of CD**			

Mild	23 (88.5%)	3 (11.5%)	-

Moderate	9 (90%)	1 (10%)	-

Severe	8 (88.9%)	-	1 (11.1%)

*Other ethnic groups 2%

At the time of diagnosis, among UC patients, 51.1% had proctitis, 22.6% left-sided disease and 26.3% extensive disease [173 out of the 240 (72.1%) UC patients had had a full colonoscopy to assess extent at the time of diagnosis), and 58.4% had mild disease, 29.5% had moderate disease, and 12.1% had severe disease. 80% of CD patients had only large bowel involvement. 2.2% of UC and 5.5% of CD patients had a positive family history. 40.4% of UC and 38.2% of CD patients had extra-intestinal manifestations. 4.7% of UC and 18.2% of CD patients had undergone surgery.

## Discussion

The present study, the first epidemiological study of IBD in this country, showed that prevalence rates were 5.3/100,000 for UC and 1.2/100,000 for CD. The incidence rates were 0.69/100,000 for UC and 0.09/100,000 for CD. The prevalence rates are low compared to Western populations [1-3], but are comparable to many, but not all, of the reported rates from Asia [14,18,19].

Shivanda *et al*. has suggested that a good epidemiological study for IBD should have a well-defined catchment area and up-to-date population data, good diagnostic facilities, uniform criteria of case definition, a common protocol and an established referral system [20]. Our study satisfied several of the above criteria. The two districts selected for the study have well defined catchment areas for the regional hospitals. Only permanent residents of the districts were included in the survey. As the most urbanised and developed districts in the country, demographic data are up-to-date, staffing and diagnostic facilities in hospitals among the best in the country. It is therefore unlikely that there is significant migration to other parts of the country for employment or health care. The high literacy rate of the population would also positively impact on health seeking behaviour [15]. Based on the results of the telephone interview of doctors in the smaller hospitals, it was reasonable to assume that most if not all IBD patients in the catchment area would be referred to specialists for investigation and treatment. The need to select only hospitals staffed by specialists would have improved the accuracy and uniformity of the diagnosis of IBD, especially because bowel infections are common in countries such as ours, and may trigger and alter the course of underlying IBD [21,22]. However, there are limitations; being a hospital based study the survey was limited to symptomatic IBD patients seeking medical care, and direct data collection was limited to patients seeking health care in hospitals. Furthermore, patients who may have sought alternative forms of treatment would have been excluded. These limitations would have led to underestimation of the true incidence and prevalence of IBD in the districts.

We observed several differences in the clinical characteristics of IBD in our patients compared to Western patients. These may well be due to IBD in Asians being genetically and phenotypically different to IBD in Western populations. There was an overall female predominance for UC (F: M = 1.5:1) but no racial differences in prevalence. This is in contrast to reports from Western and most Asian countries where there is no sex difference. The mean age at diagnosis of our patients was slightly older than that observed in Western [3] and some other Asian studies [9-13]. This may be due to late presentation at the specialist hospital, but could also reflect milder disease in this cohort. IBD was uncommon among our children, unlike in Western series. Many Western studies have found a so-called 'second peak' of prevalence for UC in the over-65s. Our patient population showed peak prevalence in the fourth decade. The apparent bimodal age distribution (Figure 2) is in agreement with Western studies [23], but is difficult to draw firm conclusions on because of the relatively small number of UC patients and even smaller number of CD patients in our study. A family history for both UC and CD was uncommon, and may indicate a lesser genetic predisposition to the disease than in Western populations, but time trend studies of the prevalence of IBD are necessary to investigate this further. Most of our patients with UC had mild, distal or left sided disease clinically and endoscopically, and responded well to medical treatment, with only a few patients requiring surgical intervention. Many of our observations are similar to those in previous reports of UC from the Asian region [7,24,25].

## Conlusion

The prevalence of UC and CD in this hospital based study was low compared to Western populations, but similar to some in Asia. Many features of IBD were similar to IBD in the West, but there were notable differences: there was a female preponderance for UC although there were no racial differences; IBD commonly presented in the fourth decade of life and was uncommon in children; only few of our patients had a family history; and most patients with UC had mild, distal or left sided disease. Investigating these differences as IBD emerges in developing countries may give further clues to its aetio-pathogenesis.

## Competing interests

The authors declare that they have no competing interests.

## Authors' contributions

MAN, APdeS, ARW, and HJdeS, conceptualized and designed the study, analyzed and interpreted the data. HGAKD, HADMPA, NMMN, SKRP, DNS, RLS, SR, ASD acquired data, and assisted in the analysis and interpretation of the data. MAN, APdeS, ARW and HJdeS drafted the initial version of the manuscript, and HGAKD, HADMPA, NMMN, SKRP, DNS, RLS, SR, ASD critically revised the manuscript. All authors read and approved the final version of the manuscript.

## Acknowledgements

The authors wish to thank Professor KI Deen and Drs JVS Aryasinghe, KVU Kalubowila, S Kumaresena and CA Jayasuriya for help with the study.

## Pre-publication history

The pre-publication history for this paper can be accessed here:

http://www.biomedcentral.com/1471-230X/10/32/prepub
